# H_2_S-Induced Sulfhydration: Biological Function and Detection Methodology

**DOI:** 10.3389/fphar.2017.00608

**Published:** 2017-09-06

**Authors:** Da Zhang, Junbao Du, Chaoshu Tang, Yaqian Huang, Hongfang Jin

**Affiliations:** ^1^Department of Pediatrics, Peking University First Hospital Beijing, China; ^2^Key Laboratory of Molecular Cardiology, Ministry of Education Beijing, China; ^3^Department of Physiology and Pathophysiology, Peking University Health Science Center Beijing, China

**Keywords:** H_2_S, sulfhydration, protein, biological function, detecting method

## Abstract

At appropriate concentrations, hydrogen sulfide, a well-known gasotransmitter, plays important roles in both physiology and pathophysiology. Increasing evidence suggests that modifying thiol groups of specific cysteines in target proteins via sulfhydration or persulfidation is one of the important mechanisms responsible for the biological functions of hydrogen sulfide. A variety of key proteins of different cellular pathways in mammals have been reported to be sulfhydrated by hydrogen sulfide to participate and regulate the processes of cell survival/death, cell differentiation, cell proliferation/hypertrophy, cellular metabolism, mitochondrial bioenergetics/biogenesis, endoplasmic reticulum stress, vasorelaxtion, inflammation, oxidative stress, etc. Moreover, *S*-sulfhydration also exerts many biological functions through the cross-talk with other post-translational modifications including phosphorylation, *S*-nitrosylation and tyrosine nitration. This review summarizes recent studies of hydrogen sulfide-induced sulfhydration as a posttranslational modification, an important biological function of hydrogen sulfide, and sulfhydrated proteins are introduced. Additionally, we discuss the main methods of detecting sulfhydration of proteins.

## Introduction

Hydrogen sulfide, a “superstar” gasotransmitter in the gaseous signal molecule family, has been found involved in various physiologic and pathophysiologic processes since the end of the last century. The role of H_2_S in the nervous, cardiovascular, digestive, and respiratory systems was examined, and the existence of endogenous H_2_S was verified. To understand how endogenous H_2_S regulates various cellular processes, researchers identified that H_2_S was involved in a post-translational modification, called *S*-sulfhydration, of a large number of proteins (Mustafa et al., 2009; Paul and Snyder, 2012). A variety of key proteins of different cellular pathways in mammals are sulfhydrated by H_2_S to regulate and affect the processes of cell survival/death, cell differentiation, cell proliferation/hypertrophy, cellular metabolism, mitochondrial bioenergetics/biogenesis, ER stress, vasorelaxtion, inflammation, oxidative stress, for example.

This review summarizes recent studies of H_2_S-induced sulfhydration as a post-translational modification that plays vital roles in diverse physiologic and pathophysiologic processes. Additionally, we discuss methods to detect sulfhydration of proteins.

## Properties of H_2_S and *S*-Sulfhydration, and Formation Process of *S*-Sulfhydration

H_2_S has some properties different from other gasotransmitters. The most typical difference is its dissociation ability. Its pKa is 6.77; under normal conditions, such as aqueous solutions at pH 7.4, over three quarters of H_2_S are dissociated to HS-anion and only 20% not dissociated, even though the concentration of S^2-^ is critically low. The H_2_S pool is believed to consist of H_2_S, HS^-^ and S^2-^. Protein persulfidation, or protein *S*-sulfhydration is regarded as one of the important molecular mechanisms by which H_2_S plays various biological effects. More accurately, this difference is mainly reflected in the modification of cysteine residues from the -SH to -SSH group. The -SH and -SSH groups differ significantly in properties. As compared with corresponding thiols (-SH), hydropersulfides (-SSH) have a stronger nucleophilic ability, for greater chemical reactivity. When pH is under physiological conditions, because of the lower pKa, hydropersulfides exhibit stronger acidity and will become more active hydrogen donors then thiols (Paul and Snyder, 2012). Another significant difference is the bond dissociation energy of S-H in RSSH or RSH: the former is 70 kcal/mol and the latter is 92 kcal/mol (Benson, 1978). Therefore, perthiyl radicals (RSS •) are more stable than thiyl radicals (RS •) which can be a very efficient antioxidant stress factor.

**FIGURE 1:**
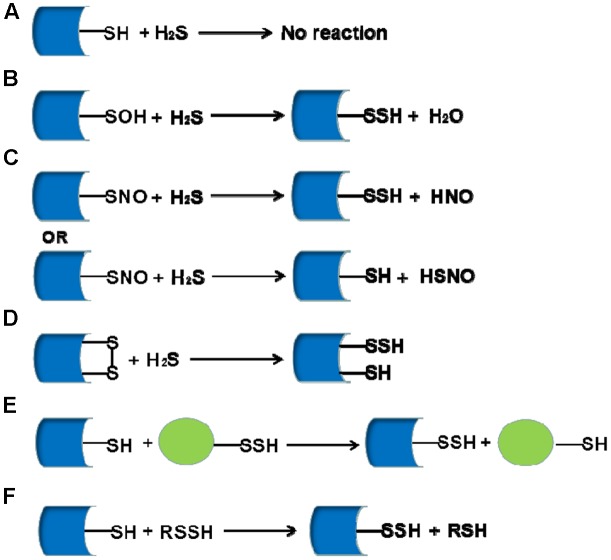
The mainly proposed formation processes for sulfhydrated proteins. **(A)** H_2_S can not react with protein thiols directly; **(B)** H_2_S can react with sulfenic acids for sulfhydration formation; **(C)** H_2_S can react with *S*-nitrosated for sulfhydration formation depending on the environment; **(D)** H_2_S reacts with cysteine disulfides (-S-S) for sulfhydration formation; **(E,F)** Persulfides can be a carrier of sulfhydration and act as the processes of displacement reaction.

In recent years, the interests of people on the biological function of sulfhydrated protein has been growing ceaselessly, but the number of the research which addressed on mechanism for the formation process of sulfhydrated proteins is still small. Here we showed some main formation processes of *S*-sulfhydrated modification which is believed to occur possibly in the following cases: (1) although protein thiols do not react with H_2_S directly, it can react with sulfenic acids; (2) H_2_S can react with *S*-nitrosated cysteines leading to the formation of HSNO or nitroxyl (HNO); (3) H_2_S can react with cysteine disulfides (-S-S) for sulfhydration formation; (4) reaction between oxidized sulfide species such as polysulfides and cysteine thiols; (5) persulfides play as carriers and engage in “*trans-S*-sulfhydration” reaction (**Figure 1**).

## Sulfhydration Mediates H_2_S-Induced Biological Function

**FIGURE 2:**
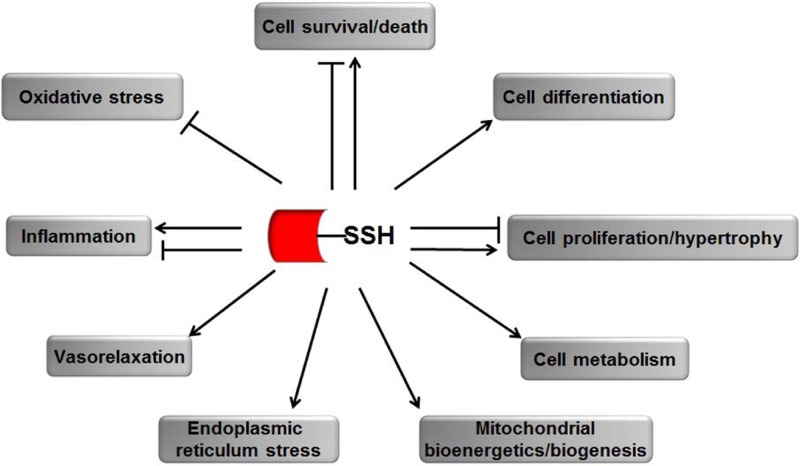
The biological functions of *S*-sulfhydration by H_2_S. → means stimulating effect, whereas ⊣ means inhibitory effect.

*S*-sulfhydrated modification as a new post-translational modification is involved in many physiological and pathological processes. After *S*-sulfhydrated, proteins would change their original function, serving as important switchers or regulators. We summarize some literatures on *S*-sulfhydrated modification targets in recent years and elucidate the important biological function of sulfhydration modification in many physiological and pathophysiological processes (**Figure 2** and **Table 1**).

**Table 1:** Examples of sulfhydrated proteins listed in alphabetical order.

Protein modified	Sites	Protein activity	Functions	Reference
AR	C611, C614	↓	Inhibits AR-DNA binding activity and AR dimerization, therefore suppresses proliferation of prostate cancer cells	Zhao et al., 2014b
ATP5A1	C244, C294	↑	Maintains ATP synthase in a physiologically activated state, supporting mitochondrial bioenergetics	Módis et al., 2016
Caspase 3	C163	↓	Prevents against neuronal ischemic/reperfusion injury	Marutani et al., 2015
eNOS	C443	↑	Promotes eNOS phosphorylation, inhibits its S-nitrosylation and increases eNOS dimerization	Altaany et al., 2014
GAPDH	C150	↑	N/A	Mustafa et al., 2009
	C156, C152	↓	N/A	Jarosz et al., 2015
GATA3	C84/182,C84/248	↓	Controls the differentiation of splenocytes and regulates the developments of allergic asthma	Wang et al., 2017
IRF-1	C53	↑	Maintains mitochondrial DNA replication	Li and Yang, 2015
Keap1	C151	↓	Activates Nrf2 signaling, attenuates oxidative stress and delays cellular aging in mouse embryonic fibroblasts	Yang et al., 2013
	C151	↓	Activates Nrf2 signaling, and attenuates oxidative stress induced by high glucose plus ox-LDL in macrophage	Xie et al., 2016
	C226, C613	↓	Activates Nrf2 signaling, protects cells from oxidative stress	Hourihan et al., 2013
Kir6.1 subunit of K_ATP_	C43	↑	Mediates the cholinergic vasorelaxation and hyperpolarization	Mustafa et al., 2011
LDHA	C163	↑	Stimulates mitochondrial respiration of the colon cancer line HCT116 and promote cell proliferation	Untereiner et al., 2017
MEK1	C341	↑	Facilitates the translocation of phosphorylated ERK into nucleus, activates PARP-1, and then increases the DNA damage repair	Zhao et al., 2014a
p65 subunit of NF-κB	C38	↑	Suppresses apoptosis induced by TNF-α in liver and macrophage physiologically	Sen et al., 2012
		↓	Inhibits ox-LDL-induced macrophage inflammation	Du et al., 2014
p66Shc	C59	↓	Inhibits H_2_O_2_ -induced mitochondrial reactive oxygen species production	Xie et al., 2014
Parkin	C59, C95, C182	↑	Enhances ubiquitination in the neuron cell lines and reduces cell death in the Parkinson disease’s cell model	Vandiver et al., 2013
PC	C265	↑	Stimulates gluconeogenesis	Ju et al., 2015
PTEN	C71, C124	↑	Inhibits the *S*-nitrosylation of PTEN	Ohno et al., 2015
PTP1B	C215	↓	Inhibits the dephosphorylation of PERK, and then promotes PERK activation during the response to endoplasmic reticulum stress	Krishnan et al., 2011
PP1c	C127	↓	Increases the phosphorylation of eIFα and induces a transient adaptive reprogramming of global mRNA translation	Yadav et al., 2017
PPARγ	C139	↑	Increases glucose uptake and lipid storage in adipocyte	Cai et al., 2016
RAGE	C259, C301	↓	Prevents the neural cell against RAGE-mediated pathological effects including oxidative stress and aging	Zhou et al., 2017
Runx2	C123, C132	↑	Promotes osteoblast differentiation and maturation	Zheng et al., 2017
Sp1	C68, C755	↑	Upregulates expression of VEGFR-2 and neuropilin-1, thereby enhances VEGF-induced endothelial responses	Saha et al., 2016
	C664	↓	Suppress expression and activity of KLF5, thereby prevents myocardial hypertrophy	Meng et al., 2016
SUR1 subunit of K_ATP_	C6, C26	↑	Activates K_ATP_ channel	Jiang et al., 2010
SUR2B subunit of K_ATP_	C24, C1455	↑	Reduces the tyrosine nitration of Kir6.1	Kang et al., 2015
TRPV6	C172, C329	↑	Induces Ca^2+^ influx in BMMSCs, and maintains BMMSC self renewal and osteogenic differentiation	Liu et al., 2014


*Effect of *S*-sulfhydration on the activity of different proteins and come-off. ↑ Denotes activation, ↓ denotes restrain, N/A denotes not mentioned. This table does not include the target protein in which specific sulfhydrated cysteine is not demonstrated. AR, androgen receptor; ATP5A1, α-subunit of ATP synthase; eNOS, endothelial NO synthase; GAPDH, glyceraldehyde 3-phosphate dehydrogenase; GATA3, GATA binding protein 3; IRF-1, Interferon regulatory factor 1; Keap1, Kelch-like ECH-associated protein 1; Nrf2, transcription factor nuclear factor erythroid 2–related factor 2; ox-LDL, oxidized low-density lipoprotein; K_ATP_, ATP sensitive potassium channel; LDHA, lactate dehydrogenase a; MEK1, map kinase kinase; ERK, extracellular signal regulated kinase; PARP-1, poly(ADP-ribose)polymerase-1; NF-κB, nuclear factor-κB; PC, pyruvate carboxylase; PTEN, phosphatase and tensin homolog deleted on chromosome ten; PTP1B, protein tyrosine phosphatase 1B; PP1c, Protein phosphatase-1; PPARγ, peroxisome proliferator-activated receptor γ; RAGE, receptor for advanced glycation endproducts; Runx2, runt-related transcription factor 2; Sp1, specificity protein 1; VEGFR, receptor of vascular endothelial growth factor; KLF5, Krüppel-like factor 5; SUR, sulfonylurea receptor; TRPV6, transient receptor potential cation channel subfamily V member 6; BMMSC, bone marrow mesenchymal stem cells.*

### *S*-Sulfhydration and Cell Survival/Death

Apoptosis or programmed cell death is a physiological process that is highly regulated by cells or tissues themselves for various biological processes. GAPDH is among the first proteins found to be modified by *S*-sulfhydration in the history of post-translational modification by H_2_S (Mustafa et al., 2009). It plays a significant role in regulating both cell survival and apoptotic death (Colell et al., 2009; Nicholls et al., 2012). GAPDH is an important redox-sensitive protein, the activity of which is largely affected by its highly reactive cysteine residue (Cys). The change in cysteine thiol helps GAPDH translate to the nucleus, where it promotes the degradation of nucleoproteins, inducing cell apoptosis (Hara et al., 2005). Mustafa et al. (2009) showed that H_2_S could uniquely *S*-sulfhydrate GAPDH at Cys150 under physiological conditions, thereby enhancing GAPDH catalytic activity. The results *in vivo* also showed that GAPDH activity was reduced by 25–30% in CSE^-/-^ mice compared with the wild type mice (Mustafa et al., 2009). Another group also confirmed that GAPDH could be *S*-sulfhydrated by endogenous H_2_S (Zhang et al., 2014). On the contrary, Jarosz et al. (2015) discovered that polysulfides inactivated the reduced purified GAPDH by 42% through *S*-sulfhydration on the Cys156. Moreover, polysulfides further decreased the activity of C156S mutant GAPDH via *S*-sulfhydration on the Cys 152, suggesting *S*-sulfhydration on the Cys 156 and Cys 152 inactivated GAPDH (Jarosz et al., 2015). Thus, modification of *S*-sulfydration may regulate GAPDH function, which controls cell apoptosis.

The DNA damage repair is an important response to maintain genomic stability, which is the basis for normal cell development and functions. Poly(ADP-ribose)ation mediated by PARPs is one of the important cellular responses to DNA damage (Audebert et al., 2004). Zhao et al. (2014a) found that H_2_S activated PARP1 and prevented DNA damage in endothelial cells and fibroblast. The protective effect of H_2_S involved ERK phosphorylation and nuclear translocation followed *S*-sulfhydrating map kinase kinase 1 (MEK1) at cysteine 341 (Zhao et al., 2014a). Mutation of cysteine 341 in MEK1 blocked the H_2_S-induced PARP1 activation, which further supported the major role of H_2_S *S*-sulfhydration in the DNA damage repair.

*S*-sulfhydrations of nuclear factor-κB (NF-κB) p65, parkin and caspase 3 were also involved in the anti-apoptotic/pro-survival effects of H_2_S. Sen et al. (2012) demonstrated that H_2_S could modify NF-κB p65 at Cys 38 thiol, enhance the binding of sulfhydrated p65 to its co-activator ribosomal protein S3, and promote the transcription of anti-apoptotic genes such as Bcl-XL and cIAP2. In cystathionine γ- lyase knock out (CSE^-/-^) mice, anti-apoptotic function and *S*-sulfhydration of NF-κB was significantly abolished, which strengthened the pro-survival role of H_2_S (Sen et al., 2012). Parkin is an E3 ubiquitin ligase which participated in the regulation of protein degradation and exerted an important neuroprotective effect. Vandiver et al. (2013) revealed that H_2_S enhanced parkin activity via sulfhydration on the Cys59, Cys95 and Cys182 sites, and then prevented cell death in the cellular models of PD. Furthermore, a marked decrease in the parkin sulfhydration in PD brain was observed, suggesting that sulfhydration of parkin is essential for neuron survival (Vandiver et al., 2013). Marutani et al. (2015) found that thiosulfate, an oxidation product of H_2_S, directly inhibited caspase 3 activity through sulfhydration at Cys163, decreased neuronal cell apoptosis, and therefore prevented against neuronal ischemic/reperfusion injury.

### *S*-Sulfhydration and Cell Differentiation

Differentiation from multipotent stem cell to terminal tissue-specific cell is important for the physiological development and pathophysiological tissue repair. In the previous studies, H_2_S was reported to regulate the differentiation of BMMSCs, periodontal ligament stem cells, neural stem cells, osteoclast and osteoblast (Wang et al., 2013; Gambari et al., 2014; Liu et al., 2014; Su et al., 2015; Zheng et al., 2017). The mechanisms involved the control of Ca^2+^ transient receptor potential cation channels, PKC/ERK-mediated Wnt/β-catenin signaling, ERK, Nrf2, Akt and Runx2 pathways by H_2_S. Liu et al found that H_2_S could sulfhydrate TRPV6 at Cys172 and Cys329 in the BMMSC, induce Ca^2+^ influx in BMMSCs, and maintain BMMSC self renewal and osteogenic differentiation. Simultaneously, sulfhydration of TRPV3 at Cys131 and sulfhydration of TRPM4 at Cys168 might be also involved in the regulatory effect of H_2_S on the osteogenic differentiation (Liu et al., 2014). Zheng et al. (2017) demonstrated that H_2_S promoted osteoblast differentiation and maturation through sulfhyating Runx2 at Cys 123 and Cys 132, which caused transactivation of Runx2.

In addition to the differentiation of stem cells, the differentiation of naïve immune cells is also important for the body homeostasis. GATA-3 is a transcription factor which controls the differentiation of naïve immune cells, demonstrated by inducing Th0 cell differentiation toward Th2 cell subtype, and promotes type-2 immune response. Wang et al found that H_2_S inhibited the transcriptional activity of GATA-3 through sulfhydrating its Cys84/182 or Cys84/248 sites, promoted the splenocyte differentiation of protective type-1 cytokine-generating cells and suppressed their differentiation toward type-2 cytokine-generating cells. Therefore, age-dependent endogenous H_2_S generation was correlated with the development of airway inflammation in the allergic asthma (Wang et al., 2017).

### *S*-Sulfhydration and Cell Proliferation/Hypertrophy

Excessive proliferation is the main feature of neoplastic disease. Modulatory effect of H_2_S on the tumor cell proliferation is different depending on tumor types. Zhao et al. (2014b) found that CSE expression was decreased in both LNCaP-B and prostate cancer tissues. H_2_S inhibited cell proliferation of both LNCaP and LNCaP-B. Moreover, forced expression of CSE restored the sensitivity of LNCaP-B cells to androgen antagonists. Mechanistically, they demonstrated that AR mediated the abovementioned effect of H_2_S evidenced by the facts that H_2_S sulfhydrated AR at Cys 611 and 614, destroyed the functional zinc finger structure in the AR, then inhibited the transcriptional activity of AR, and accordingly suppressed the proliferation of prostate cancer (Zhao et al., 2014b). On the contrary, Untereiner et al. (2017) revealed that endogenous H_2_S production and expression of its generating enzyme CBS in colon cancer cells were upregulated. H_2_S promoted the proliferation of colon cancer cell HCT116 via sulfhydrating LDHA at Cys163 to enhance its activity.

Inadequate proliferation of endothelial cell is one of the vital characterizations of endothelial dysfunction, which resulted in vascular injury diseases. Saha et al. (2016) found that cystathionine β-synthase (CBS)-derived H_2_S maintained the VEGF-dependent cellular response including VEGF-dependent proliferation resulting from increased VEGFR-2 and neuropilin-1 expression in endothelial cell, mediated by the *S*-sulfhydration of the transcription factor Sp1 at residues Cys68 and Cys755 and therefore enhanced the transcriptional activity of Sp1 (Saha et al., 2016). This study confirmed the deficiency of CBS/H_2_S-mediated protein *S*-sulfhydration in the development of vascular dysfunction.

Myocardial hypertrophy is a major adaptive response of cardiomyocyte when it meets various stimulators. The KLF5 was an important signaling contributed to the development of cardiac hypertrophy induced by angiotensin II (Shindo et al., 2002). Meng et al. (2016) found that H_2_S donor GYY4137 decreased KLF5 promoter activity, reduced KLF5 mRNA expression, inhibited transcriptional activity of KLF5, and therefore prevented cardiomyocyte hypertrophy *in vitro* and *in vivo*. The above effects of H_2_S were mediated by its sulfhydration of Sp1 at Cys664 to block the binding of Sp1 to the KLF5 promoter (Meng et al., 2016).

### *S*-Sulfhydration and Cellular Metabolism

More and more studies confirmed that H_2_S acted as an important regulator of lipid and glucose metabolism. It was reported that H_2_S modulated the adipogenesis, lipolysis, apolipoprotein biosynthesis, glucose utilization, glucogenesis, and insulin resistance, etc. The impairment of endogenous H_2_S generation and function was the important pathogenesis of dyslipidemia and/or hyperglycemia-related diseases. Cai et al found that H_2_S promoted the triglyceride accumulation in adipocyte differentiation, increased the adipocyte number in mice fed with a high-fat diet for 4 weeks and alleviated insulin resistance of adipose tissues but did not increase the obesity of mice fed with high-fat diet for 13 weeks simultaneously. The mechanism by which H_2_S changed glucose into triglyceride storage in adipocytes was associated with the facts that H_2_S induced *S*-sulfhydration of PPARγ at Cys 139, increased its nuclear translocation and DNA binding activity, and promoted adipogenesis gene expression (Cai et al., 2016). Ju et al. (2015) explored the role of *S*-sulfhydration modified by CSE-derived H_2_S in the regulation of glucogenesis. The data showed that H_2_S donor or overexpression of CSE induced PC sulfhydration, enhanced PC activity, therefore promoted glucose production in liver cells. Furthermore, site-directed mutation at Cys 265 blocked H_2_S-induced PC sulfhydration and activity (Ju et al., 2015). Additionally, sulfhydration of peroxisome proliferator-activated receptor-γ coactivator-1α, fructose-1,6-bisphosphatase and glucose-6-phosphatase was also involved in the regulation of hepatic glucose production (Untereiner et al., 2016b).

### *S*-Sulfhydration and Mitochondrial Bioenergetics/Biogenesis

The steady state of mitochondria is very important during a cell life and is often metobolism-related, including ATP synthesis and processes that regulate cell growth and death. In the last few years, increasing evidence showed that H_2_S could stimulate mitochondrial bioenergetics and act as a mitochondrial protectant (Jornayvaz and Shulman, 2010; Bartosz et al., 2014). PPARγ coactivator-related protein (PPRC) has positive effect in maintaining the stability of cell energy metabolism and normal cell viability. PPRC could be *S*-sulfhydrated by H_2_S, and the level was lower in untreated CSE–knockout hepatocytes, which regulated cell energy homeostasis under physiological conditions as well as mitochondrial bioenergetics (Untereiner et al., 2016a). H_2_S can also induce a *S*-sulfydration of α subunit of ATP synthase (ATP5A1) at Cys244 and Cys294, which maintains ATP synthase activation under physiological condition, thereby supporting mitochondrial bioenergetics (Módis et al., 2016). Li and colleagues confirmed the role of H_2_S in maintaining mitochondrial DNA replication and mitochondrial marker gene expression. They revealed that H_2_S sulfhydrated IRF-1 at Cys 53, enhanced its binding with the Dnmt3a promoter, reduced Dnmt3a expression, and induced mitochondrial transcription factor A promoter demethylation and therefore promoted mitochondrial DNA replication (Li and Yang, 2015).

### *S*-Sulfhydration and Endoplasmic Reticulum Stress (ERS)

Endoplasmic reticulum is composed of a membrane in eukaryotic cells and an important organelle for protein synthesis, folding and secretion. External or internal environment changes will lead to ERS. The PTP family is widely recognized as a group of fundamental enzymes that control various biological processes, such as cell–cell communication, cell growth, division and differentiation (Sato et al., 1998). PTP-1B is a vital member of the PTPs protein family; it locates in the cytoplasmic face of ER and plays a key role in ER signaling (Bellomo et al., 2016). PTP-1B loses its enzymatic activity when H_2_S S-sulfhydrates its active-site Cys215 residue both *in vivo* and *in vitro*, thereby promoting the activity of protein kinase RNA-like ER kinase and restoration of ER homeostasis during the response to ERS (Krishnan et al., 2011). Phosphorylation of eIF2α, resulting in inhibition of global protein synthesis, is one of the key biochemical steps for ERS. Yadav et al found that H_2_S could inhibit PP1c via sulfhydration at Cys127, block the dephosphylation of eIF2α and therefore regulate the ERS (Yadav et al., 2017). Some new pathways by which H_2_S controls ERS have been recently disclosed, including Akt-heat shock protein 90 pathway (Xie et al., 2012), brain-derived neurotrophic factor-TrkB pathway (Wei et al., 2014), silent mating type information regulator 2 homolog 1 (Li et al., 2014), and Src pathway (Ying et al., 2016), etc. However, most of these studies focused on turnon/off of the protein but not the *S*-sulfhydrated protein, nor the specific cysteine affected by H_2_S stimulation. Therefore, further studies are needed to elaborate the mechanism by which H_2_S inhibits ERS.

### S-Sulfhydration and Vasorelaxtion

As one of the important biological functions induced by H_2_S, vasorelaxation of H_2_S and its mechanisms have been extensively studied (Hosoki et al., 1997). A series of target proteins including ion channels and second messengers were found to be involved in the control of vessel tone by H_2_S. Since *S*-sulfhydration was demonstrated, the molecular mechanisms responsible for H_2_S-induced vasodilation were understood significantly. K_ATP_ are composed of pore-forming subunits and regulatory subunits, including Kir6.x (Kir6.1 or Kir6.2), and SURx (SUR1, SUR2A or SUR2B), which mediated the H_2_S-induced vasorelaxation in aorta and mesenteric artery (Zhao et al., 2001; Cheng et al., 2004). Jiang et al. (2010) found that Cys6 and Cys26 in the extracellular loop of rat vascular SUR1(rvSUR1) were target of H_2_S-induced *S*-sulfhydration. H_2_S opened the K_ATP_ channel to exert a vasorelaxation via *S*-sulfhydration of K_ATP_ channel (Jiang et al., 2010), while a research by Mustafa and colleagues revealed that H_2_S induced hyperpolarization in endothelial cells mediated by the opening of Kir 6.1 subunit of K_ATP_ channel via its sulfhydration at Cys43 (Mustafa et al., 2011). Additionally, *S*-sulfhydration of endothelial intermediate conductance potassium channel, small conductance potassium channel and TRPV4 might be in part due to vascular relaxation induced by H_2_S (Mustafa et al., 2011; Naik et al., 2016). Sun et al found that H_2_S could increase intracellular cGMP level via sulfhydrate phosphodiesterase 5A to inhibit the cGMP degradation (Sun et al., 2017). Moreover, Yu et al. (2017) demonstrated that *S*-sulfhydration of TRPV1 by CBS-derived H_2_S in carotid sinus facilitated carotid sinus baroreceptor sensitivity to participated the control of blood pressure.

### S-Sulfhydration and Inflammation

The relationship between H_2_S and inflammation is complex. The anti-inflammatory effect of H_2_S was reported in carrageenan-induced paw edema (Zanardo et al., 2006), colitis (Fiorucci et al., 2007; Wallace et al., 2009), synovitis (Ekundi-Valentim et al., 2010), monoarthritis (Andruski et al., 2008), atherosclerosis (Wang et al., 2009), ischemia-reperfusion injury (Zuidema et al., 2010), cigarette smoke-induced pulmonary injury (Chen et al., 2011; Han et al., 2011) and diabetic wound healing (Zhao et al., 2017), etc. NF-κB signaling is widely known as an important pathway in regulating inflammatory response. Du et al. (2014) found that H_2_S inhibited macrophage inflammation induced by oxidized low-density lipoprotein via the sulfhydration of NF-κB p65 at Cys38, thereby restraining NF-κB p65 phosphorylation, nuclear translocation, DNA binding activity and the recruitment to monocyte chemotactic protein-1 promoter. In an experimental model of colitis, endogenous H_2_S synthesis was upregulated and played a protective role due to the activation of K_ATP_ via the *S*-sulfhydration of its subunit SUR2B (Wallace et al., 2009; Gade et al., 2013). In addition to the abovementioned target proteins, there are many other proteins or pathways involved in the anti-inflammatory effect of H_2_S. However, whether H_2_S sulfhydrates those proteins to inhibit the inflammatory response remains unclear.

On the other hand, Bhatia et al. (2005) demonstrated that the treatment with DL-propargylglycine, a CSE inhibitor, significantly reduced the severity of pancreatitis and lung injury induced by caerulein. Similarly, in caecal-ligation and puncture-induced sepsis mice model, CSE gene deletion alleviated the liver and lung injury and reduced inflammation along with the activation of ERK1/2 and NF-κB pathway (Gaddam et al., 2016). Those results suggested that H_2_S played a role as pro-inflammatory cytokines. Whether H_2_S is an anti-inflammatory or pro-inflammatory agent is controversial (Whiteman and Winyard, 2011). Therefore, more in-depth studies are needed for broader conclusive answers to elaborate the relationship between H_2_S and inflammation (Wallace et al., 2012).

### *S*-Sulfhydration and Oxidative Stress

Numerous experimental results show that the oxidative stress sensor protein Keap1 and Nrf2 are closely related to the oxidative stress injury and the antioxidant response (Uesugi et al., 2017; Wasik et al., 2017). Previous studies suggested that H_2_S played an important role in protecting against oxidative stress by enhancing Nrf2 nuclear translocation and initiating antioxidant response in ischemia-reperfusion injury (Calvert et al., 2009; Guo et al., 2014; Shimada et al., 2015), diabetes-accelerated atherosclerosis (Xie et al., 2016) and high salt-induced renal injury (Huang et al., 2016). Moreover, Nrf2 activation mediated the inhibitory effect of H_2_S on the oxidative stress-induced cell senescence (Yang et al., 2013). Regarding the molecular mechanism by which H_2_S activated Nrf2-initiating antioxidant response, Yang et al. (2013) and Xie et al. (2016) elucidated that NaHS could *S*-sulfhydrate Keap1 at Cys151, which promoted the dissociation of Nrf2 from Keap1, while Hourihan et al. (2013) found that H_2_S inactivated Keap1 through sulfhydrating Keap1 at Cys226/613 site.

P66Shc, an upstream activator of mitochondrial redox signaling, plays a pivotal role in the regulation of intercellular redox homeostasis. Phosphorylation of p66Shc at Ser36 was regarded as a key step to fire the reactive oxidative species production. Xie et al. (2014) discovered that H_2_S could sulfhydrate p66Shc at Cys59 to inhibit p66Shc phosphorylation, reduce its translocation to mitochondria, block the mitochondrial reactive oxidative species production, and thereby protect neuronal cells against oxidative stress-induced injury. Activation of the RAGE is the key element in the development of the chronic oxidative stress-induced cytotoxicity. Zhou et al found that the treatment of NaHS reduced H_2_O_2_ –induced RAGE dimerization, shortened the half-life of RAGE, decreased the plasma membrane abundance of RAGE and therefore prevented neuron SH-SY5Y cells from cytotoxicity. Mechanistically, cys259 and cys310, which mediated the formation of intermolecular disulfide bond in the RAGE, were verified to be the direct target sites of H_2_S *S*-sulfhydration (Zhou et al., 2017). Those abovementioned studies are important for better determining the mechanism by which H_2_S exerts the protective role in the oxidative stress-induced diseases.

### *S*-Sulfhydration and Other Post-translational Modification

The relationship of *S*-sulfhydration and phosphorylation: Xie et al. (2014) and Du et al. (2014) found that H_2_S-induced *S*-sulfhydration could inhibit phosphorylation of p66Shc and NF-κB p65, and decreased their activity. On the contrary, Altaany et al. (2014) found that H_2_S enhanced eNOS activity by promoting phosphylation of eNOS, which resulted from H_2_S-induced *S*-sulfhydation of eNOS at Cys 443.

The relationship of *S*-sulfhydration and *S*-nitrosylation: For a long period, researches have established that nitric oxide (NO) can act as an important regulator in diverse cell signaling pathway via *S*-nitrosylation happened at cysteine residue of target protein (Jaffrey et al., 2001). Under basal conditions, 10–25% of proteins in liver total proteins were *S*-sulfhydrated, while 1–2% of proteins were *S*-nitrosylated (Jaffrey et al., 2001; Mustafa et al., 2009). They have some similar chemical properties. For example, it was proposed that the two modifications preferentially occured at low pKa Cys residues of the protein (Lu et al., 2013). Many proteins have been confirmed to be controlled by both *S*-nitrosylation and *S*-sulfhydration such as GAPDH, parkin, eNOS, PPARγ, PTP1B, PTEN, p65, SUB2B and etc (Chung et al., 2004; Hara et al., 2005; Chen et al., 2008; Mustafa et al., 2009; Vandiver et al., 2013; Altaany et al., 2014; Cao et al., 2015; Ohno et al., 2015; Cai et al., 2016). In most cases, *S*-sulfhydration and *S*-nitrosylation exert opposite effects. For instance, the glycolytic activity of GAPDH is inhibited by *S*-nitrosylation (Hara et al., 2005), whereas *S*-sulfhydration increases its activity about sevenfold (Mustafa et al., 2009). Parkin activity is decreased when *S*-nitrosylated (Chung et al., 2004) but increased when *S*-sulfhydrated (Vandiver et al., 2013). *S*-nitrosylation of PPARγ at Cys139 inhibits PPARγ transcription activity (Cao et al., 2015), but *S*-sulfhydration of PPARγ at the same residue enhances its activity (Cai et al., 2016). Similarly, *S*-nitrosylation of PTP1B at Cys 215 prevents it from H_2_O_2_-induced inactivation (Chen et al., 2008), but *S*-sulfhydration of PPARγ at the same residue inhibits its activity (Krishnan et al., 2011). Furthermore, H_2_S-induced *S*-sulfhydration could directly inhibit *S*-nitrosylation of eNOS to prevent eNOS from inactivation (Altaany et al., 2014). Ohno and colleagues found that *S*-sulfhydration of PTEN at Cys71 and Cys124 by CBS/H_2_S could prevent its *S*-nitrosylation induced by NO and restore NO-caused PTEN inactivation under physiological conditions (Ohno et al., 2015). However, Sun et al demonstrated that an increased *S*-nitrosylation level contributed to the additive myocardial postconditioning protection with H_2_S donor plus NO donor (Sun et al., 2016). Therefore, the interaction between S-nitrosylation and *S*-sulfhydration might be one kind of complicate communications between H_2_S-excited signaling and NO-induced signaling.

The relationship of *S*-sulfhydration and tyrosine nitration: The tyrosine nitration is a posttranslation modification by peroxynitrite and other reactive nitrogen species which happened at free tyrosine or protein tyrosine residues. Tyrosine nitration is regarded to partly mediate the cytotoxicity of reactive nitrogen species (Franco and Estévez, 2014). Kang et al. (2015) found that *S*-sulfhydration of SUR2B subunit of K_ATP_ channel at Cys 24 and Cys 2455 residues caused by H_2_S donor NaHS could prevent tyrosine nitration of Kir 6.1, another subunit of K_ATP_ channel, and K_ATP_ inactivation induced by peroxynitrite. Also, NaHS could decrease the calcium channel nitration and prevent the inhibitory effect of peroxynitrite in CaCl_2_-induced isolated mouse ileum contraction (Kang et al., 2015). The study demonstrates a new mechanism responsible for cytoprotective effect of H_2_S in reactive nitrogen species-induced injury and disease.

## Methods For *S*-Sulfhydrated Protein Detection

**FIGURE 3:**
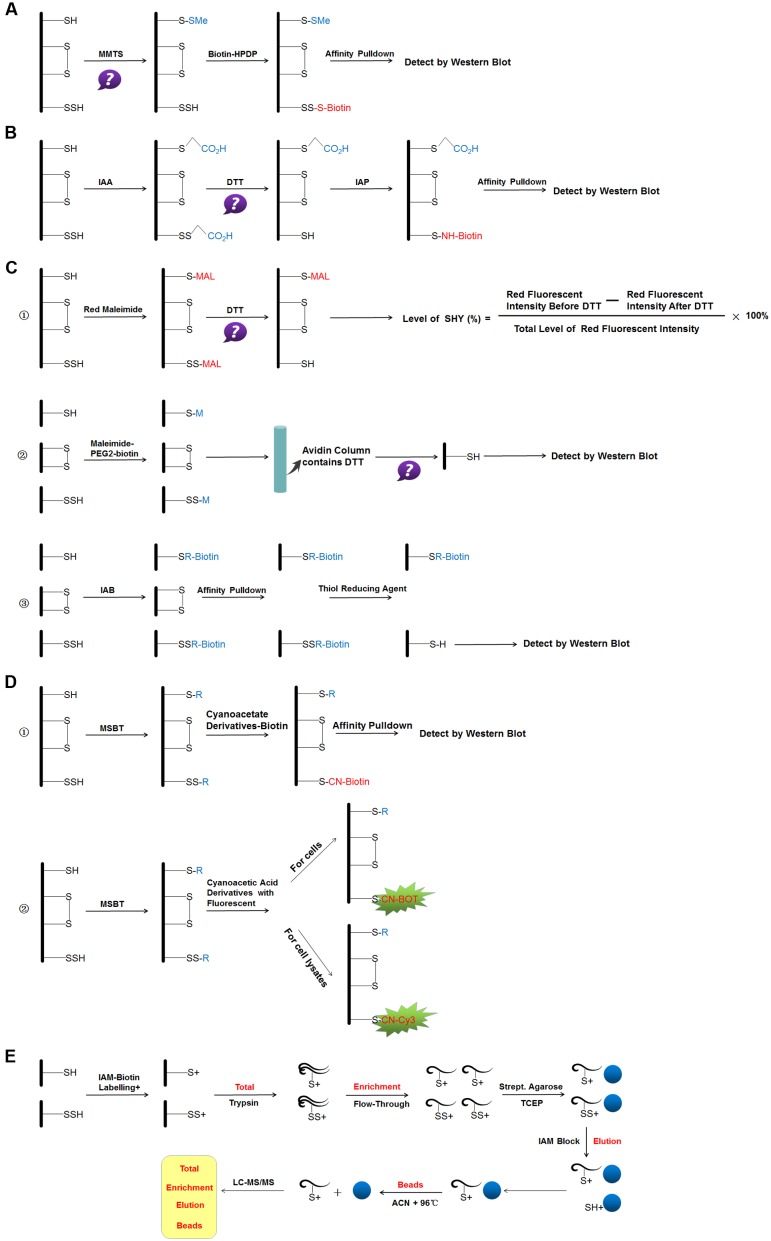
Reaction schemes of different methods for *S*-sulfhydraion detection. **(A)** biotin-switch assay; **(B)** cysteinyl labeling assay; **(C)** ➀ the maleimide assay, ➁ improved method of maleimide assay (Biotin-Thiol Assay) and ➂ protein persulfide detection protocol (ProPerDP); **(D)** ➀ tag-switch assay and ➁ new tag-switch assay; **(E)** mass spectrometry assay. MMTS: *S*-methyl methanethiosulfonate, Biotin-HPDP: *N*-[6-(biotinamido)hexyl]-3′-(2′-pyridyldithio) propinamide, IAA, Iodoacetic acid; DTT, dithiothreitol; IAP, Iodoacetamide-linked biotin; IAB, Iodoacetyl-PEG_2_-Biotin; MSBT, methylsulfonyl benzothiazole; CN-BOT, Cyanoacetic acid derivatives with the fluorescent BODIPY moiety; CN-Cy3, Cyanoacetic acid derivatives with the fluorescent Cy3-dye; IAM-Biotin, Iodoacetyl-PEG2-Biotin; TCEP, Tris(2-carboxyethyl)phosphine; IAM, Iodoacetamide; ACN, Acetonitrile; LC-MS/MS, Liquid chromatography and mass spectrometry.

Establishing a detection method of protein *S*-sulfhydration has remained challenging for a long period. Scientists have investigated methods of detection to distinguish the persulfide group and free thiols. We here summarize the *S*-sulfhydration detection methods and discuss their advantages and the possible limitations in the experimental process (**Figure 3**).

### Biotin-Switch Assay

The original method of *S*-sulfhydrated protein detection, named biotin-switch assay, was described by Mustafa et al. (2009). The authors simplified the original method of detecting nitrosylation for the specific cysteine thiol modification. In the first step, the thiol-blocking reagent MMTS is used to react with the -SH group. In the subsequent step, MMTS was removed by acetone, and persulfides were labeled with biotin-HPDP in dimethyl sulfoxide. Then, biotinylated proteins were pulled down by streptavidin-agarose beads, and then were washed with HENS buffer. For the last step, the biotinylated proteins were eluted by SDS-PAGE sample buffer and examined by western blot analysis. Mustafa and colleagues suggested that up to 25% proteins, especially in liver were sulfhydrated under basal conditions (Mustafa et al., 2009). *S*-sulfhydration is substantially more prevalent than nitrosylation and represents a previously unappreciated, major post-translational modification. Although this method is the first to identify *S*-sulfhydrated proteins, it has limitations. MMTS has been widely used in *S*-nitrosylation detection. The method for *S*-sulfhydrated group detection is based on the fact that the *S*-sulfhydrated protein does not react with MMTS as the prerequisite. However, in 2013, a study found that the -SH group could react with MMTS directly, which suggested that the biotin-switch assay was based on an incorrect chemical premise (Pan and Carroll, 2013). The authors explained this phenomenon from the perspective of two mechanisms. First, MMTS may not block and alkylate all of the free thiols; the unblocked free thiols will then react with the pyridyldisulfide biotin reagent. Second, in the presence of a large number of free sulfhydryl groups unblocked in the first step, biotin labeling may be achieved by stepwise thiol–disulfide exchange. Hence, all the different situations that may result in the false-positive results of this method can be considered as not all the free sulfhydryl groups being completely blocked during the MMTS labeling step. Although this method has been in doubt and some other new methods were found later, many research teams also evaluated this method as sensitive and selective. New proteins that can be *S*-sulfhydrated were found by this original method (Módis et al., 2016; Li et al., 2016).

### Cysteinyl Labeling Assay

In 2011, a new method based on a completely opposite principle of chemistry was used. The authors proposed that a new kind of thiol-blocking reagent, named IAA, would react with both *S*-sulfhydrated protein and free thiols (Krishnan et al., 2011). In the first step, desalting columns were pre-processed by IAA-free lysis buffer and then cell lysates were slowly passed through it. In the next step, DTT was applied to IAA-cleared lysates. During this phase, the authors proposed that the alkylated Cys residue, whether persulfide or another reversibly oxidized form, would reduce back to the thiolate state. So, at a last step, IAP was used to label the particular cysteine. The objection to this method is that it cannot distinguish persulfides from intramolecular, intermolecular and *S*-nitrosothiols, which will also be cleaved by DTT.

### The Maleimide Assay, Biotin-Thiol-Assay and Protein Persulfide Detection Protocol

Sen et al. (2012) suggested a third method based on the chemical characteristics that *N*-ethyl maleimide (thiol-blocking reagent) would block both free thiol and persulfide. Cy5-conjugated maleimide was used in the first step and DTT in the second. The principle of this method is that the fluorescence signal decreases when the sample contains persulfides, and the decreased ratio of the fluorescence signal is the quantitative index (Sen et al., 2012). The limitation of this method is that it cannot be used widely for proteomic analysis.

A study in 2015 improved this method. The investigators made some changes to the experiment, named BTA (Gao et al., 2015). The authors used NM-Biotin to alkylate both cysteine residues or sulfhydrated cysteine in the first step. In the subsequent step, the avidin column was purified and eluted with DTT for cleaving the disulfide bonds; however, the biotin tag was still left bound to the column. The eluate from the column is further examined by western blot analysis. Furthermore, Dóka and colleagues described another new method named ProPerDP in early 2016. In the first step, IAB was used to alkylate both thiol and persulfide functional groups, but IAB would not discriminate and react with oxidized Cys residues in the original sample. In the second step, steptavidin-coated magnetic beads were used to pull down with alkylated proteins, with oxidized Cys residues maintained in the supernatant. In the last step, reducing buffer was used to re-suspend purified beads for cleaving the original persulfides as thiols selectively from steptavidin-coated magnetic beads, for the next step of determining persulfide proteins (Dóka et al., 2016). The abovementioned two methods still have limitations. First, some proteins contain both sulfhydryl groups of the -SSH group and other non-persulfidated Cys residues. Therefore the protein that contains the -SSH group cannot cleave off from streptavidin beads in the last step, which would lead to false-negative signals. Second, the structure of disulfide bonds in intermolecular protein may result in false-positive persulfidated extra Cys residues on the polypeptide chains, which may also lead to false-positive signals. Third, to overcome the above-mentioned problem, a possible approach is to digest the alkylated protein before the pulldown step, because ensuring that the disulfide and free Cys moieties or persulfidated Cys would maintain the same form and in the same peptide is difficult.

### Tag-Switch Assay

Zhang et al. (2014) proposed a new method to detect protein *S*-sulfhydrated that is based on the different physical and chemical properties between -SH and -SSH groups, named Tag-switch Assay (Zhang et al., 2014). In the first step, a thiol-blocking reagent reacts with both free thiol and persulfide. The authors proposed that, compared with common disulfides in proteins, the disulfide bond in persulfide adducts might have a stronger reactivity to nucleophilicity. Then, a new tag-switching reagent was used to label persulfide protein only. This method has been accepted by some research teams (Park et al., 2015; Zhou et al., 2017). The main challenge for this new method is to ensure that the disulfide linkage in the -SSH group can express a specific and suitable nucleophile and then distinguish persulfide from free thiols. Treating cell lysates with dimedone was suggested to avoid any potential cross reaction (Park et al., 2015).

Wedmann et al. (2016) proposed to improve the original tag-switch method. The biotinylated cyanoacetic acid tag was used for the assay in the original tag-switch method, which requires western blot and unique antibodies for analyzing the final results. To increase the sensitivity, the authors synthesized two new kinds of cyanoacetic acid derivatives with the fluorescent BODIPY moiety (CN-BOT) for labeling cells and Cy3-dye (CN-Cy3) for labeling cell lysates. The improved tag-switch method for persulfide detection provides a new starting point for future researches to elucidate the actual mechanisms for H_2_S signaling pathway.

However, soon, some other chemists described the deficiencies of the tag-switch method. First, MSBT cannot penetrate the cell membranes, which poses an obstacle for the detection of persulfide in living cells. Besides that, in the “switching” step, methyl cyanoacetate was proposed to uniquely cut off MSBT-labeled dialkyl disulfides among the protein disulfide moieties. Nevertheless, selectivity was specific: it was only assessed on glutathionylated BSA and on the *N*-tert-butyloxycarbonyl derivatized cystine (Nagy, 2013). Although the tag-switch method for persulfide detection is adopted by increasing numbers of research teams, as a new methodology, it also needs more trials to verify the true reliability and for comparison with previous methods.

### Mass Spectrometry Assay

Most recently, Longen et al. (2016) described details of mass spectrometry-based workflow for determining *S*-sulhydrated proteins and their sites. The proteins collected from cells were precipitated with trichloroacetic acid. The protein thiols and persulfides were labeled with IAMBio. After digestion of the proteins (Total), single peptides containing labeled persulfides or cysteines were concentrated and separated from non-cysteine peptides (flow through) through using streptavidin agarose beads. After several washes, persulfide-containing peptides were eluted by using TCEP with no effect on thiol-containing peptides. The subsequent accessible cysteines were labeled with iodoacetamide (Elution). As a control, labeled thiol peptides remaining on the beads were eluted by using 10 mM TCEP and 80% acetonitrile (Beads). Samples of the total, flow-through, elution and bead fraction were subjected to liquid chromatography and mass spectrometry and the peptides were identified by using PEAKS 7.0 proteomics software (Longen et al., 2016).

## The Controversy of Sulfurated Modification

Lately, investigators have discovered that a substance called polysulfide has cytoprotective effects also through mechanisms involving sulfurated modification of target proteins. The oxidation state of sulfur atom in thiol and H_2_S is -2. Atoms do not react with each other under the same oxidative state; therefore, H_2_S cannot sulfurate cysteine residues theoretically. The internal sulfur of H_2_S_n_ is 0. Therefore, it reacts with thiol readily (Kimura, 2015). However, cysteine residues are oxidized to two main forms —cysteine sulfenic acid or cysteine *S*-nitrosothiol — under oxidative conditions. H_2_S shows a stronger ability to sulfurate these oxidized thiols than H_2_S_n_ (Kabil and Banerjee, 2014; Kimura, 2015). Therefore, when we verify target proteins that are thiolated, we should further verify and distinguish whether the sulfurated modification is due to H_2_S or H_2_S_n_. However, sulfurated modification, whether due to H_2_S or H_2_S_n_, is now well established. At present, the focus of the debate may lie in the specific process of -SSH, but it does not affect consensus on the important role of -SSH in various pathological and physiological processes. The balance between H_2_S and H_2_S_n_ plays a key role in controlling cellular metabolism.

## Conclusion and Perspectives

With increasing studies concerning the effect of H_2_S on phenotype in physiological and pathological processes, the mechanism by which H_2_S functions in different signaling pathways via *S*-sulfhydration has gradually been recognized. *S*-sulfhydration is a new post-translational modification of proteins by H_2_S. From the beginning of this century, different research groups globally have found that many proteins can be modified by H_2_S via *S*-sulfhydration, but there are still a considerable amount of results did not link to the sulfhydration. Furthermore, we still need to elucidate the sites of *S*-sulfhydration that H_2_S acts on. Though some new views point out that polysulfides exert a cytoprotective effect, a lot of studies on the biological function of H_2_S remain. It is gratifying that a large number of laboratory experiments and clinical trials have revealed H_2_S to have a positive effect on the regulation of physiological and pathological processes and the inhibition of the disease progression. With better understanding of more proteins to be post-modified by H_2_S via *S*-sulfhydration, the biological protective effect of H_2_S will be well recognized.

## Author Contributions

DZ, JD, and HJ provided the overall concept and framework of the manuscript. DZ and YH researched and identified appropriate articles. DZ participated in writing the manuscript. JD, HJ and CT revised the manuscript. All authors approved the final version of the manuscript.

## Conflict of Interest Statement

The authors declare that the research was conducted in the absence of any commercial or financial relationships that could be construed as a potential conflict of interest.

## Abbreviations

AR: androgen receptor
ATP: adenosine triphosphate
ATP5A1: α subunit of ATP synthase
BMMSCs: bone marrow mesenchymal stem cells
biotin-HPDP: N6-(biotinamido)hexyl]-3′-(2′-pyridyldithio) propionamide
BSA: bovine serum albumin
BTA: biotin-thiol-assay
CBS: cystathionine β-synthase
CSE: cystathionine γ-lyase
Cys: cysteine
DTT: dithiothreitol
eNOS: endothelial nitric oxide synthase
ER: endoplasmic reticulum
ERK: extracellular signal-regulated kinase
GAPDH: Glyceraldehyde 3-phosphate dehydrogenase
GATA-3: GATA binding protein 3
HNO: nitroxyl
H_2_S: hydrogen sulfide
HSNO: thionitrous acid
K_ATP_: ATP sensitive potassium channels
Keap1: Kelch-like ECH-associated protein 1
KLF5: Krüppel-like factor 5
IAA: iodoacetic acid
IAB: biotin-tagged alkylating agent
IAMBio: iodoacetyl-PEG2-Biotin
IAP: iodoacetamide-linked biotin probe
IRF-1: interferon regulatory factor-1
LDHA: lactate dehydrogenase A
LNCaP: androgen-dependent prostate cancer cells
LNCaP-B: antiandrogen-resistant prostate cancer cells
MEK: map kinase kinase
MMTS: *S*-methyl methanethiosulfonate
MSBT: methylsulfonyl benzothiazole
NaHS: sodium hydrosulfide
NF-κB: nuclear factor-κB
NM-Biotin: maleimide-PEG2-biotin
NO: nitric oxide
Nrf2: nuclear factor erythroid 2–related factor 2
PC: pyruvate carboxylase
PD: Parkinson’s disease
PARP: poly(ADP-ribose)ation polymerases
PP1c: protein phosphatase-1c
PPARγ: peroxisome proliferator activated receptor γ
PPRC: proliferator-activated receptor-γ coactivator-related protein
ProPerDP: protein persulfide detection protocol
PTEN: phosphatase and tensin homolog deleted on chromosome ten
PTP: protein tyrosine phosphatase
RAGE: receptor for advanced glycation endproducts
Runx2: Runt-related transcription factor 2
Sp1: specificity protein 1
TRPV6: transient receptor potential cation channel subfamily V member 6
TCEP: tris(2-carboxyethyl)phosphine
VEGF: vascular endothelial growth factor
VEGFR: VEGF receptor
